# Characterization of visceral branch geometry in a four-branch off-the-shelf device for endovascular repair of paravisceral and thoracoabdominal aortic aneurysms

**DOI:** 10.1016/j.jvscit.2026.102332

**Published:** 2026-06-03

**Authors:** Fatima Mustansir, Muhammad Zeeshan, Ryan Wahidi, J. Westley Ohman, Luis A. Sanchez, Zachary J. Wanken

**Affiliations:** Division of Vascular Surgery, Washington University in St Louis, St Louis, MO

**Keywords:** Aortic aneurysm, Endovascular repair, Branched and fenestrated endograft

## Abstract

**Background:**

This study aims to evaluate visceral branch geometry, including angulation, tortuosity, and diameter, of the GORE EXCLUDER thoracoabdominal branch endoprosthesis (TAMBE) in real-world utilization.

**Methods:**

All patients were identified and treated with TAMBE from June 2024 to June 2025 at a single institution. Using the first postoperative computed tomography scan, branch geometry was analyzed with three-dimensional reconstructive software including multiplanar reformats and centerline imaging. Per published guidance, centerline branch length (length 1, L1) was defined as the centerline distance from the distal edge of the portal to the end of the branch, including any covered stent but not including distal extension with an uncovered self-expanding stent. Direct length (length 2, L2) was defined as the direct distance between those two points. Tortuosity index was calculated as the ratio of L1/L2. Branch angle was measured between the portal and a line tangential to the distal end point of the branch. Branch diameter was measured at the distal end point of the stent. We used paired *t*-test for between-branch comparisons and two-sample *t*-test for comparisons by sex and between complex abdominal aortic aneurysm and thoracoabdominal aortic aneurysm.

**Results:**

A total of 29 patients were treated with TAMBE during the study period. The majority of patients were male (n = 18, 62.1%) and white (n = 27, 93.1%). The mean age was 72.6 ± 7.2 years (range, 51-81 years). A total of 25 patients had postoperative computed tomography available for analysis, with 93 visceral branches analyzed (celiac artery [CA]: n = 20, superior mesenteric artery: n = 25, right renal artery [RRA]: n = 24, and left renal artery [LRA]: n = 24). Pairwise comparisons revealed significant differences between the CA, superior mesenteric artery, and renal arteries in terms of all geometric parameters including L1, L2, tortuosity index, and distal branch diameter, but no significant difference between RRA and LRA. Between-group comparisons showed a greater degree of CA angulation in females (142 ± 23° in females vs 113 ± 23° in males, *P* = .02) and significantly greater distal renal artery branch diameters in males (RRA: 6.2 ± 0.6 mm in males, 5.4 ± 0.6 mm in females, *P* = .02; LRA: 6.2 ± 0.5 mm in males, 5.7 ± 0.5 mm in females, *P* = .03). Between-group comparisons for the remaining branch parameters did not show a significant difference.

**Conclusions:**

We demonstrate that geometric parameters of visceral branches in TAMBE vary significantly, whereas differences in terms of sex and aneurysm extent are less common. Evaluating the impact of these differences on long-term branch outcomes may help guide device selection and optimize patient outcomes.

**Article Highlights**

• **Type of Research:** Single-center retrospective imaging study evaluating postoperative visceral branch geometry after implantation of the GORE EXCLUDER thoracoabdominal branch endoprosthesis
• **Key Findings:** Among 25 patients (93 visceral branches), celiac branches were significantly shorter, renal branches were more tortuous and smaller in diameter than mesenteric branches, and women had smaller renal branch diameters than men. Branch geometry was otherwise similar between complex abdominal and thoracoabdominal aneurysms.
• **Take Home Message:** Visceral branch geometry after thoracoabdominal branch endoprosthesis implantation varies systematically by target vessel but minimally by aneurysm extent. Understanding these geometric differences may help optimize branch planning, guide adjunctive techniques, and inform future studies evaluating branch durability and target vessel patency.

Complex abdominal aortic aneurysm (CAAA) and thoracoabdominal aortic aneurysm (TAAA), which require incorporation of visceral branches into the repair, are challenging to treat. Traditional, open surgical repair requiring clamp position above the visceral vessels carries substantially more risk than repair requiring an infrarenal clamp.^1^ Higher extent thoracoabdominal repairs carry even greater morbidity and mortality.^2^ Many patients are not candidates for open repair and require an endovascular solution.^3^ Before 2024, there were no off-the-shelf devices approved for use in the United States, and repairs were limited to custom-made or physician-modified devices.^4^ Patient access was limited based on device availability and the regulatory landscape.^5^

However, the GORE EXCLUDER thoracoabdominal branch endoprosthesis (TAMBE) was approved by the US Food and Drug Administration and became commercially available in June 2024.^6^ The TAMBE device provides an off-the-shelf endovascular solution for CAAA and extent IV TAAA and incorporates four downgoing branches for incorporation of visceral vessels.^7^ Data from the device pivotal trial demonstrated a safe and effective treatment profile in the early time period, albeit with a rate of renal branch occlusion that raises some concern, particularly in patients with pararenal aneurysm.^8^ Previous studies have shown that visceral branches, which are longer, more angulated, more tortuous, and smaller in diameter, are at higher risk for branch instability, such as branch occlusion or dissociation, leading to endoleak and necessitating reintervention.^9^, ^10^, ^11^, ^12^

We therefore sought to characterize branch geometry for TAMBE repair in real-world utilization. Beyond characterization, we compared geometry between men and women and across aneurysm extent. We aim to better understand the impact of branch geometry on target vessel instability with the TAMBE device so that we can better understand the anatomic constraints of the device or postulate adjunctive branch techniques to preserve patency.

## Methods

### Patient population

We identified all patients treated with the TAMBE device at our institution from June 2024 to June 2025, the first year after its commercial availability. We collected baseline demographics as well as data on comorbid conditions and medication usage. Aneurysm extent was defined by published convention. CAAAs included juxtarenal, pararenal, and paravisceral aneurysms.^13^ TAAAs were classified as extent I, II, III, or IV based on the Crawford classification.^14^ Visceral branch geometry was measured using the first postoperative computed tomography (CT) angiography examination performed after implantation. For patients with noncontrast CT imaging alone, branch geometry was extracted based on stent architecture. Patients without postoperative imaging were excluded from our analysis.

### Characterization of branch geometry

CT imaging studies were evaluated using three-dimensional reconstructive software (Aquarius Intuition; TeraRecon, Inc) including multiplanar reformats and centerline imaging. Individual centerlines were created for each visceral branch, which ended in the celiac artery (CA), superior mesenteric artery (SMA), right renal artery (RRA), or left renal artery (LRA). We excluded branches that extended beyond these main axes. For example, a branch that extended to the common hepatic artery beyond the celiac axis proper was excluded from the analysis.

**Fig 1:**
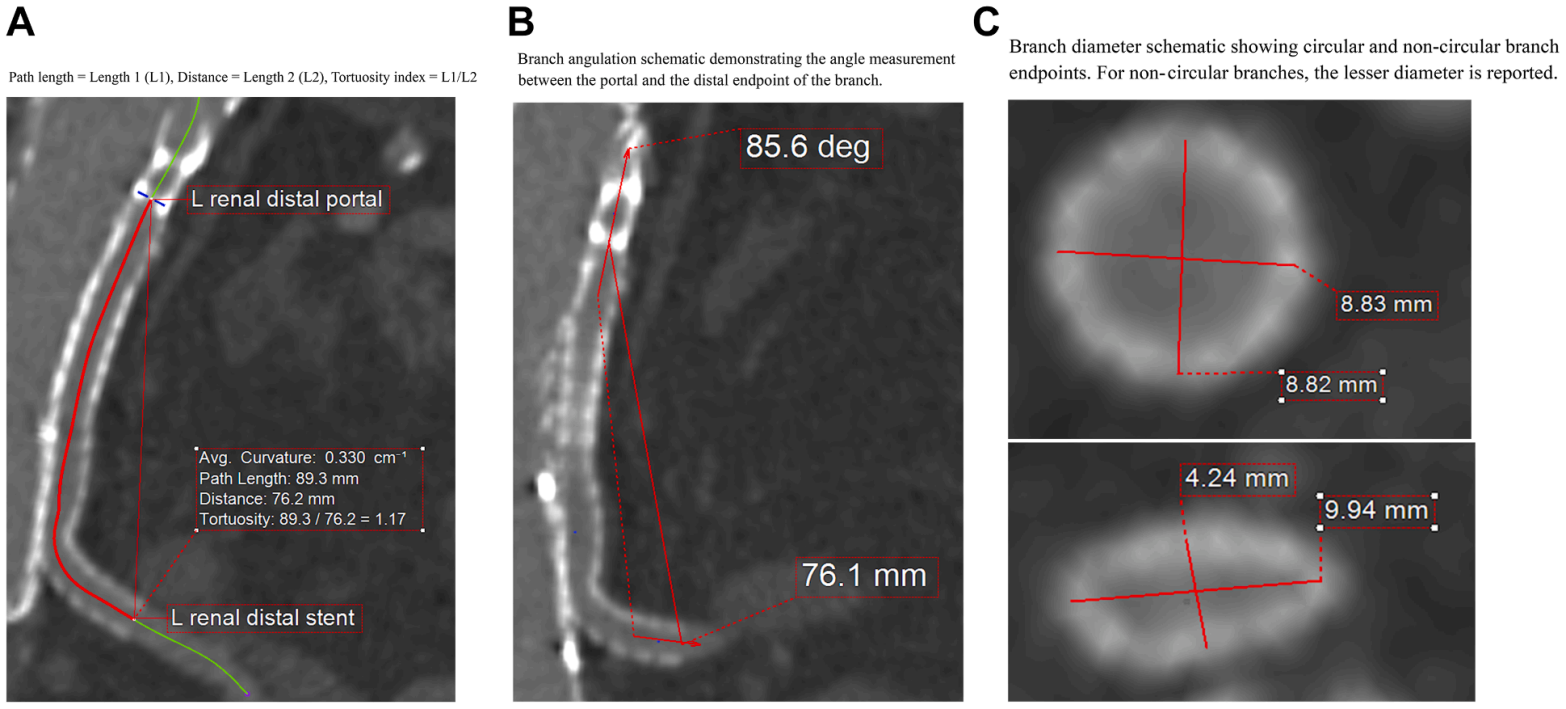
Schematic of branch measurement techniques. **A,** Path length = length 1 (L1), distance = length 2 (L2), tortuosity index = L1/L2. **B,** Branch angulation schematic demonstrating the angle measurement between the portal and the distal end point of the branch. **C,** Branch diameter schematic showing circular and noncircular branch end points. For noncircular branches, the lesser diameter is reported.

We defined lengths and tortuosity index (TI) based on published guidance (Fig 1).^15^ We defined length 1 (L1) to be the centerline distance from the distal edge of the portal to the distal edge of the covered branch and not including extension with an uncovered stent. We defined length 2 (L2) to be the direct distance between the same two points (the distal edge of the portal and the distal edge of the covered branch). We calculated TI as the ratio of centerline length to direct length (L1/L2). We measured branch angulation as the angle between the downgoing portal and a line tangential to the distal aspect of the branch. Although these two vectors were not always in the same plane, the angulation was estimated as closely as possible for geometric characterization along the vertical axis. Branch diameter was measured at the distal end point of the stent graft. For branches with noncircular geometry, the lesser diameter is reported.

### Data reporting and statistical analysis

Continuous variables (including centerline length, direct length, branch angulation, and diameter) are reported as mean and standard deviation after distributions were checked for normality. Given the left-bounded nature of TI as a ratio, values are reported as median, range, and interquartile range (IQR).

We compared parameters between individual visceral branches to evaluate for differences using paired *t*-tests for within-patient comparison. In addition, we compared branch geometry between groups based on aneurysm extent and sex using two-sample *t*-tests for centerline length, direct length, branch angulation, and diameter. We used Wilcoxon rank-sum test for TI given the non-normal distribution for that variable.

For data visualization, violin plots were used to show the weighted distributions of the data for each branch. Statistical analysis was performed using STATA 19BE (StataCorp LLC). This study was approved by the institutional review board of Washington University in St Louis, with a waiver of informed consent granted due to its retrospective design.

## Results

During the study period, a total of 29 patients were treated with the TAMBE device. A majority were male (n = 18, 62.1%) and white (n = 27, 93.1%). The mean age was 72.6 ± 7.2 years (range, 51-81 years), and comorbidities were typical of an aortic aneurysm cohort (Table I). A total of 25 patients had postoperative CT available for analysis. Of the four patients without postoperative imaging, one was lost to follow-up, one died of unclear cause, and two are doing well in follow-up with imaging obtained after data collection had been completed.

**Table I:** Patient demographics

	No. or mean ± standard deviation	Percentageor range
Age, years	72.6 ± 7.2	51-81
Sex, male	18	62.1
Body mass index, kg/m^2^	26.5 ± 6.0	18.0-34.8
Race/ethnicity		
White	27	93.1
African American	2	6.9
Diabetes mellitus	5	17.2
Coronary artery disease	8	27.6
Previous myocardial infarction	5	17.2
Congestive heart failure	10	34.5
Chronic kidney disease (≥stage 3)	10	34.5
Peripheral artery disease	5	17.2
Chronic obstructive pulmonary disease	12	41.4
Hypertension	28	96.6
Hyperlipidemia	22	75.9
Smoker (current or former)	26	96.3
Prior stroke	7	21.4
American Society of Anesthesia class		
1	0	0
2	0	0
3	12	41.3
4	17	58.6

Categorical data are shown as number (%).

**Fig 2:**
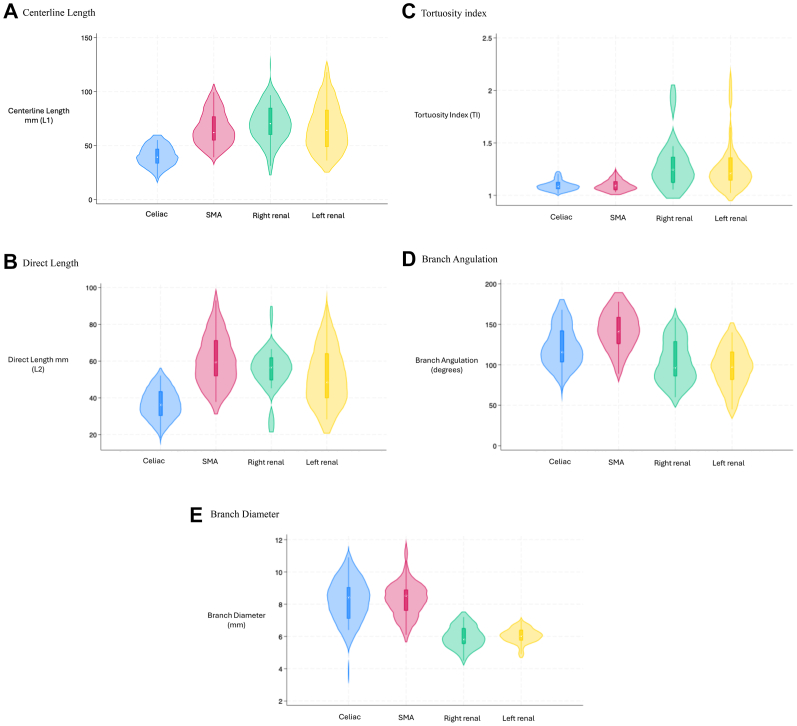
Branch characteristics. **A,** Centerline length. **B,** Direct length. **C,** Tortuosity index. **D,** Branch angulation. **E,** Branch diameter.

A total of 93 visceral branches were analyzed, with 20 branches in the CA, 25 branches in the SMA, 24 branches in the LRA, and 24 branches in the RRA. Of the remaining seven portals, 4 were embolized as part of a planned three-vessel reconstruction (n = 3 celiac, n = 1 renal), two celiac branches were extended to the common hepatic and excluded from the analysis, and one renal branch was unable to be cannulated and branched during the index operation. Branch parameters and comparisons are shown in Table II, with distributions visualized in the violin plots, as shown in Fig 2. We did not identify any branch occlusions at this time period of follow-up.

**Table II:** Branch characteristics and pairwise comparisons

	Celiac (n = 20)	SMA (n = 25)	RRA (n = 24)	LRA (n = 24)
Centerline length (L1)				
Celiac branch: mean, 40.2 ± 9.7 mm; median [IQR], 39.3 [33.5, 47.0] mm; range, 19.5-55.2 mm	–	*P* < .001	*P* < .001	*P* < .001
SMA: mean, 66.7 ± 16.4 mm; median [IQR], 62.1 [54.8, 77.0] mm; range, 39.2-99.5 mm	*P* < .001	–	*P* = .23	*P* = .57
RRA: mean, 71.3 ± 20.4 mm; median [IQR], 70.4 [60.1, 85.0] mm; range, 31.3-125.0 mm	*P* < .001	*P* = .23	–	*P* = .38
LRA: mean, 68.5 ± 23.3 mm; median [IQR], 64.2 [48.9, 83.1] mm; range, 36.2-118.0 mm	*P* < .001	*P* = .57	*P* = .38	–
Direct length (L2)				
Celiac branch: mean, 36.6 ± 8.5 mm; median [IQR], 36.1 [30.3, 43.5] mm; range, 18.1-52.1 mm	–	*P* < .001	*P* < .001	*P* < .001
SMA; mean, 60.7 ± 14.3 mm; median [IQR], 59.4 [51.8, 71.3] mm; range, 37.8-93.0 mm	*P* < .001	–	*P* = .08	*P* = .02
RRA: mean, 55.1 ± 12.1 mm; median [IQR], 56.4 [49.7, 61.9] mm; range, 25.6-85.6 mm	*P* < .001	*P* = .08	–	*P* = .27
LRA: mean, 52.4 ± 16.2 mm; median [IQR], 48.4 [39.9, 64.2] mm; range, 28.3-90.7 mm	*P* < .001	*P* = .02	*P* = .27	–
Tortuosity index				
Celiac branch: mean, 1.096 ± 0.055; median [IQR], 1.082 [1.061, 1.126]; range, 1.015-1.205	–	*P* = .43	*P* = .004	*P* = .006
SMA: mean, 1.097 ± 0.055 mm; median [IQR], 1.091 [1.052, 1.134]; range, 1.030-1.244 mm	*P* = .43	–	*P* < .001	*P* = .001
RRA: mean, 1.293 ± 0.233 mm; median [IQR], 1.241 [1.122, 1.366]; range, 1.055-1.969 mm	*P* = .004	*P* < .001	–	*P* = .55
LRA: mean, 1.325 ± 0.303 mm; median [IQR], 1.209 [1.143, 1.359]; range, 1.023-2.156 mm	*P* = .006	*P* = .001	*P* = .55	–
Branch angle				
Celiac branch: mean, 121 ± 26°; median [IQR], 116 [104, 142]°; range, 67-168 deg	–	*P* = .01	*P* = .16	*P* = .007
SMA: mean: 142 ± 25°; median [IQR], 141 [126, 159]°; range, 89-178°	*P* = .01	–	*P* < .001	*P* < .001
RRA: mean, 104 ± 27°; median [IQR], 96 [86, 129]°; range, 60-158°	*P* = .16	*P* < .001	–	*P* = .35
LRA: mean, 96 ± 27°; median [IQR], 97 [82, 116]°; range, 45140°	*P* = .007	*P* < .001	*P* = .35	–
Distal branch diameter				
Celiac branch: mean, 8.1 ± 1.6 mm; median [IQR], 8.4 [7.1, 9.1] mm; range, 3.810.9 mm	–	*P* = .68	*P* < .001	*P* < .001
SMA: mean, 8.4 ± 1.2 mm; median [IQR], 8.5 [7.6, 8.9] mm; range, 6.1-11.6 mm	*P* = .68	–	*P* < .001	*P* < .001
RRA: mean, 6.0 ± 0.7 mm; median [IQR], 5.8 [5.6, 6.5] mm; range, 4.5-7.2 mm	*P* < .001	*P* < .001	–	*P* = .59
LRA: mean, 6.0 ± 0.6 mm; median [IQR], 6.0 [5.8, 6.4] mm; range, 4.9-7.0 mm	*P* < .001	*P* < .001	*P* = .59	–

*SMA*, Superior mesenteric branch; *RRA*, Right renal branch; *LRA*, Left renal branch; *IQR*, interquartile range.

Of the 20 branches in the CA, 13 were completed with one Gore Viabahn balloon-expandable (VBX) stent graft and 7 were completed with two Gore VBX stent grafts. Of the 25 branches in the SMA, 8 were completed with one Gore VBX stent graft, 14 were completed with two Gore VBX stent grafts, 1 was completed with three Gore VBX stent grafts, 1 was completed with a Gore Viabahn distal extension to a Gore VBX stent graft, and 1 was completed with a Gore VBX stent graft with distal extension using an uncovered self-expanding stent. Of the 24 branches in the RRA, 7 were completed with one Gore VBX stent graft and 17 were completed with two Gore VBX stent grafts. Of the 24 branches in the LRA, 7 were completed with one Gore VBX stent graft, 13 were completed with two Gore VBX stent grafts, 2 branches were completed with three Gore VBX stent grafts, 1 branch was completed with a Gore Viabahn extension to a Gore VBX stent graft, and 1 branch was completed with a Gore VBX stent graft with distal extension using an uncovered self-expanding stent.

The centerline length of the CA branch was 40.2 ± 9.7 mm (range, 19.5-55.2 mm). The centerline length of the SMA branch was 66.7 ± 16.4 mm (range, 39.2-99.5 mm). The centerline length of the RRA branch was 71.3 ± 20.4 mm (range, 31.3-125.0 mm). The centerline length of the LRA branch was 68.5 ± 23.3 mm (range, 36.2-118.0 mm). Between-branch comparisons showed that the CA branch was significantly shorter in centerline length when compared with all other branches (*P* < .001 for all comparisons). Comparisons between the SMA and renal artery branches were not significantly different.

The direct length of the CA branch was 36.6 ± 8.5 mm (range, 18.1-52.1 mm). The direct length of the SMA branch was 60.7 ± 14.3 mm (range, 37.8-93.0 mm). The direct length for the RRA branch was 55.1 ± 12.1 mm (range, 25.6-85.6 mm). The direct length of the LRA branch was 52.4 ± 16.2 mm (range, 28.3-90.7 mm). Between-branch comparisons showed that the CA branch was significantly shorter in direct length when compared with all other branches (*P* < .001 for all comparisons). Comparisons also showed that the SMA branch was longer in direct length when compared with the LRA branch (*P* = .02) and not significantly different when compared with the right renal branch (*P* = .08).

The median TI of the CA branch was 1.082 (IQR, 1.061-1.126; range, 1.015-1.205). The median TI of the SMA branch was 1.091 (IQR, 1.052-1.134; range, 1.030-1.244). The median TI of the RRA branch was 1.241 (IQR, 1.122-1.366; range, 1.055-1.969). The median TI of the LRA branch was 1.209 (IQR, 1.143-1.359; range, 1.023-2.156). Comparisons showed that the higher TI of the renal branches was significantly greater than that of the mesenteric branches. Comparisons showed no significant difference in TI between the CA branch and SMA branch or in comparison of LRA to RRA.

The branch angulation of the CA branch was 121 ± 26° (range, 67-168°). The branch angulation of the SMA branch was 142 ± 25° (range, 89-178°). The branch angulation for the RRA branch was 104 ± 27° (range, 60-158°). The branch angulation of the LRA branch was 96 ± 27° (range, 45-140°). Comparisons showed that the celiac branch angulation was significantly more acute than the SMA branch (*P* = .01) and that the renal artery branch angulation was significantly more acute than the SMA branch (*P* < .001 for both left and right renal branches).

The branch diameter at the distal end point of the CA branch was 8.1 ± 1.6 mm (range, 3.8-10.9 mm). The branch diameter at the distal end point of the SMA branch was 8.4 ± 1.2 mm (range, 6.1-11.6 mm). The branch diameter at the distal end point of the RRA branch was 6.0 ± 0.7 mm (range, 4.5-7.2 mm). The branch diameter at the distal end point of the left renal artery branch was 6.0 ± 0.6 mm (range, 4.9-7.0 mm). Comparisons showed that the mesenteric branches (both CA and SMA) were significantly larger than the renal branches (both left and right, *P* < .001).

In comparison of branch characteristics between TAAA and CAAA, we did not find any significant differences for any of the measured parameters (Table III). In comparison to branch angulation between males and females, we found that the celiac branch was significantly more acutely angulated in men as compared with women and therefore had a slightly higher TI (nonsignificant, Table IV). The branch diameter was significantly smaller for both right and left renal branches in women compared with men (*P* = .02 and *P* = .03, respectively). The remainder of the comparisons did not show a significant difference.

**Table III:** Comparison by aneurysm extent

	CAAA	TAAA	*P* value
Celiac branch	n = 10	n = 10	
Centerline length (L1), mm	40.7 ± 11.3	39.6 ± 8.4	.80
Direct length (L2), mm	37.2 ± 10.4	35.9 ± 6.7	.73
Tortuosity index, median [IQR]	1.089 [1.063-1.122]	1.072 [1.058-1.131]	.88
Branch angulation, °	121.3 ± 22.0	122.3 ± 31.1	.93
Distal branch diameter, mm	8.0 ± 1.8	8.2 ± 1.4	.80
SMA branch	n = 11	n = 14	
Centerline length (L1), mm	68.2 ± 17.0	65.5 ± 16.4	.69
Direct length (L2), mm	62.9 ± 15.0	59.0 ± 14.0	.52
Tortuosity index	1.070 [1.051-1.100]	1.095 [1.052-1.145]	.38
Branch angulation, °	141.7 ± 16.9	141.7 ± 30.1	1.00
Distal branch diameter, mm	8.6 ±1.3	8.2 ± 1.2	.46
Right renal branch	n = 11	n = 13	
Centerline length (L1), mm	67.0 ± 15.7	74.8 ± 23.7	.36
Direct length (L2), mm	54.5 ± 10.2	55.6 ± 13.9	.83
Tortuosity index	1.191 [1.105-1.337]	1.259 [1.130-1.434]	.40
Branch angulation, °	115.4 ± 22.3	94.6 ± 27.9	.06
Distal branch diameter, mm	6.0 ± 0.7	5.9 ± 0.7	.60
Left renal branch	n = 11	n = 13	
Centerline length (L1), mm	64.8 ± 24.1	71.6 ± 23.1	.49
Direct length (L2), mm	55.0 ± 19.2	50.1 ± 13.6	.47
Tortuosity index	1.162 [1.130-1.247]	1.341 [1.168-1.646]	.06
Branch angulation, °	104.8 ± 20.7	88.3 ± 30.5	.14
Distal branch diameter, mm	6.1 ± 0.7	6.0 ± 0.5	.77

*CAAA*, Complex abdominal aortic aneurysm; *TAAA*, thoracoabdominal aortic aneurysm; *SMA*, superior mesenteric branch.

**Table IV:** Comparison by patient sex

	Male	Female	*P* value
Celiac branch	n = 14	n = 6	
Centerline length (L1), mm	39.2 ± 10.5	42.3 ± 8.0	.53
Direct length (L2), mm	35.1 ± 8.4	40.0 ± 8.4	.25
Tortuosity index (median [IQR])	1.094 [1.072-1.143]	1.061 [1.058-1.071]	.06
Branch angulation, °	113.1 ± 23.0	142.2 ± 22.9	.02
Distal branch diameter, mm	8.2 ± 1.6	7.9 ± 1.7	.76
SMA branch	n = 17	n = 8	
Centerline length (L1), mm	68.0 ± 17.3	63.9 ± 14.9	.57
Direct length (L2), mm	61.4 ± 15.3	59.3 ± 12.6	.74
Tortuosity index	1.095 [1.070-1.143]	1.055 [1.044-1.087]	.09
Branch angulation, °	136.4 ± 23.1	153.1 ± 25.4	.12
Distal branch diameter, mm	8.7 ± 1.0	7.8 ± 1.5	.09
Right renal branch	n = 17	n = 7	
Centerline length (L1), mm	73.4 ± 20.0	66.1 ± 22.1	.44
Direct length (L2), mm	57.3 ± 11.8	49.8 ± 12.1	.18
Tortuosity index	1.259 [1.123-1.337]	1.223 [1.120-1.469]	.97
Branch angulation, °	105.7 ± 25.4	100.3 ± 32.6	.67
Distal branch diameter, mm	6.2 ± 0.6	5.4 ± 0.6	.02
Left renal branch	n = 16	n = 8	
Centerline length (L1), mm	67.2 ± 23.4	71.1 ± 24.4	.71
Direct length (L2), mm	54.1 ±16.8	49.0 ± 15.4	.48
Tortuosity index	1.171 [1.143-1.263]	1.435 [1.197-1.763]	.11
Branch angulation, °	101.3 ± 22.8	85.0 ± 33.6	.17
Distal branch diameter, mm	6.2 ± 0.5	5.7 ± 0.5	.03

*SMA*, Superior mesenteric branch.

## Discussion

In this study of visceral branch geometry in TAMBE repair, we identified many differences between branches and patient populations. We found that the celiac branch was significantly shorter in both centerline distance and direct distance compared with the other visceral branches. The renal artery branches were significantly more tortuous than the CA and SMA branches as measured by the TI. The SMA branch was the straightest, whereas the celiac and renal branches had significantly more acute angulations. Between-group comparisons showed smaller branch diameter in women as compared with men, particularly for the renal branches. However, branch characteristics were similar between patients with TAA and those with CAAA.

Published description of TAMBE geometry is limited, which served as part of the impetus for the present study. In comparing our results with the feasibility study, however, we do note that our SMA branches are 30% longer on average and renal branches are 10% to 15% longer despite our celiac branches being slightly shorter.^7^ However, it is worth noting that the feasibility study included a device configuration with upgoing renal portals, which may explain the shorter renal branch length. The difference in SMA branch length is more difficult to elucidate, especially as the feasibility study included celiac branch placement for all patients, whereas our study included several patients without celiac branch incorporation due to chronic occlusion. The branches in our study are larger in diameter, averaging about 1 mm larger in the visceral branches and slightly larger in the renal branches as well.

The broad base of literature on branched and fenestrated endovascular repair of CAAA and TAAA has consistently shown several risk factors for target vessel instability. Longer, smaller, and more angulated branches are at higher risk of instability. Therefore, it was not surprising when the TAMBE pivotal trial demonstrated a low but concerning rate of renal branch instability with 1-year freedom from target vessel instability of 90.8% and 89.8% in the right and left branches, respectively.^6^ The length, angulation/tortuosity, and size are all likely factors in long-term outcomes for branches. As we found, the significantly smaller renal branch size for women as compared with men raises questions about differential long-term patency based on sex, especially as the cohort in the pivotal study was composed of 82% males.

Our goal with this project is to inform device selection for individual patient anatomy and inform the use of adjunctive techniques to preserve branch patency. Adjunctive branch techniques, such as the utilization of a balloon-expandable stent-graft proximally and a self-expanding stent-graft distally, have been well described.^16^ However, the Gore VBX stent-graft (W.L. Gore & Associates) was specifically designed for use with TAMBE and is structured with independent stents to facilitate curvature of the branches.^17^ We found the devices to take curves well and only required extension with a self-expanding stent for end point dissection. We anecdotally and infrequently identified compression of the celiac branch at the median arcuate ligament but did not find branch compression issues otherwise.

Our study has limitations. We report the branch geometry from a single center, which may not be generalizable to the population at large. The geometric parameters described in our article provide a limited sample of factors that affect the flow dynamics and long-term stability/patency of branches. These parameters were measured after branch placement, and comparison to preprocedural branch anatomy is beyond the scope of this article. We also do not know the effect on long-term patency and outcomes for the branches and plan to evaluate that in follow-up work in the future.

## Conclusions

In this article, we characterized the geometry of visceral branches in TAMBE and described the differences between visceral and renal branches. In addition, we identified differences in branch geometry between males and females as well as between CAAA and TAAA. Evaluating the impact of these differences on long-term branch outcomes may help guide device selection to optimize patient outcomes.

## Funding

None.

## Disclosures

Z.J.W. reports serving as a consultant for Terumo Aortic and acts as the site principal investigator for the TAMBE Post-Approval Study (PAS). L.A.S. reports serving as a consultant for W.L. Gore & Associates. JWO reports serving as consultants for W.L. Gore & Associates, Cook Medical, and Terumo Aortic and discloses intellectual property holdings with Globus Medical. The remaining authors have no conflicts of interest to disclose.

## References

[1] Varkevisser R.R.B., de Guerre L.E.M.V., Swerdlow N.J. (2020). The impact of proximal clamp location on peri-operative outcomes following open surgical repair of juxtarenal abdominal aortic aneurysms. Eur J Vasc Endovasc Surg.

[2] Moulakakis K.G., Karaolanis G., Antonopoulos C.N. (2018). Open repair of thoracoabdominal aortic aneurysms in experienced centers. J Vasc Surg.

[3] Huang Y., Colglazier J., Pochettino A. (2025). Treatment trends and outcomes of endovascular versus open thoracoabdominal aortic aneurysm repair - a single-center comparative cohort study. Ann Surg.

[4] Tsilimparis N., Melo R.G.E., Tenorio E.R. (2024). Multicenter study on physician-modified endografts for thoracoabdominal and complex abdominal aortic aneurysm repair. Circulation.

[5] Finnesgard E.J., Jones D.W., Beck A.W. (2025). Trends and outcomes over time with fenestrated and branched endovascular aortic repair in the United States aortic research consortium. J Vasc Surg.

[6] Farber M.A., Matsumura J.S., Han S. (2024). Early outcomes from the pivotal trial of a four-branch off-the shelf solution to treat complex abdominal and type IV thoracoabdominal aortic aneurysms. J Vasc Surg.

[7] Oderich G.S., Farber M.A., Silveira P.G. (2019). Technical aspects and 30-day outcomes of the prospective early feasibility study of the GORE EXCLUDER Thoracoabdominal Branched Endoprosthesis (TAMBE) to treat pararenal and extent IV thoracoabdominal aortic aneurysms. J Vasc Surg.

[8] Farber M.A., Han S., Makaroun M.S. (2025). One-year outcomes from the pivotal trial of a four-branch off-the-shelf solution to treat pararenal and extent IV thoracoabdominal aortic aneurysms. J Vasc Surg.

[9] Chaikof E.L., Fillinger M.F., Matsumura J.S. (2002). Identifying and grading factors that modify the outcome of endovascular aortic aneurysm repair. J Vasc Surg.

[10] Piazza M., Squizzato F., Xodo A., Gubert A., Grego F., Antonello M. (2021). Effect of branch length and tortuosity on the outcomes of branched endovascular repair of thoracoabdominal aneurysms using self-expandable bridging stent graft. J Vasc Surg.

[11] Becker D., Sikman L., Ali A. (2024). Analysis of target vessel instability in fenestrated endovascular repair (f-EVAR) in thoraco-abdominal aortic pathologies. J Clin Med.

[12] Becker D., Sikman L., Ali A., Prendes C.F., Stana J., Tsilimparis N. (2024). The impact of target vessel anatomy and bridging stent geometry on branched endovascular aortic repair outcomes. Eur J Vasc Endovasc Surg.

[13] Chaikof E.L., Blankensteijn J.D., Harris P.L. (2002). Reporting standards for endovascular aortic aneurysm repair. J Vasc Surg.

[14] Crawford E.S., Crawford J.L., Safi H.J. (1986). Thoracoabdominal aortic aneurysms: preoperative and intraoperative factors determining immediate and long-term results of operations in 605 patients. J Vasc Surg.

[15] Oderich G.S., Forbes T.L., Chaer R. (2021). Reporting standards for endovascular aortic repair of aneurysms involving the renal-mesenteric arteries. J Vasc Surg.

[16] Gallitto E., Faggioli G., Vacirca A. (2025). Hybrid stent graft technique in bridging hostile renal arteries in thoraco-abdominal branched endografting. Eur J Vasc Endovasc Surg.

[17] Motta F., Parodi F.E., Knowles M. (2021). Performance of Viabahn balloon-expandable stent compared with self-expandable covered stents for branched endovascular aortic repair. J Vasc Surg.

